# Editorial: Nuclear receptors in health and disease

**DOI:** 10.3389/fendo.2025.1744402

**Published:** 2025-11-27

**Authors:** Natalia E. Santucci, Laura Solt, Vinicius de Frias Carvalho

**Affiliations:** 1 Laboratorio de Estudios en Tuberculosis, Instituto de Inmunología Clínica y Experimental de Rosario (IDICER)-Consejo Nacional de Investigaciones Científicas y Técnicas (CONICET)-Universidad Nacional de Rosario (UNR), Rosario, Argentina; 2 Facultad de Ciencias Médicas, Universidad Nacional de Rosario (UNR), Rosario, Argentina; 3 Department of Immunology and Microbiology, Herbert Wertheim UF Scripps Institute for Biomedical Innovation & Technology, Rosario, Argentina; 4 Instituto Nacional de Ciência e Tecnologia em Neuroimunomodulação (INCT-NIM), Instituto Oswaldo Cruz, Fundação Oswaldo Cruz, Rio de Janeiro, Brazil

**Keywords:** nuclear receptor (NR), immunoendocrine response, immunomodulation, transcription factor, signaling

Nuclear receptors (NRs) are master regulators of cellular function, converting endocrine and metabolic signals into precise transcriptional programmes and rapid signalling events. As fundamental conductors of processes such as proliferation, immune response, and metabolic homeostasis (1), their pivotal role in systemic communication means their dysregulation is a direct driver of pathology, making them indispensable targets for modern therapeutics (2).

NRs help define cellular identity by orchestrating the gene expression programmes that guide development and maintain homeostasis. They further coordinate organ-specific functions by modulating complex gene networks across diverse cell types. However, this transcriptional regulation also extends into disease, where it is often co-opted into compensatory—and frequently deleterious—feedback loops, highlighting their dual role in both homeostasis and disease (1). This duality is eloquently exemplified in the immune system. Here, NRs transduce signals from a diverse portfolio of lipophilic ligands into precise genomic instructions. By binding to DNA, assembling regulatory complexes, and altering chromatin architecture (3), they direct cellular fate. Yet, this central role carries an inherent vulnerability: when dysregulated, NRs can propel the development of modern diseases such as cancer, autoimmune disorders, and chronic inflammation, and older diseases, including infectious diseases (1, 3).

NR activity is governed by a dual-input system: lipophilic ligands and post-translational modifications. Ligands, which range from endocrine hormones to dietary lipids, can cross the plasma membrane and induce a conformational change in the receptor’s ligand-binding domain. This structural shift is the central event that alters the receptor’s subcellular localisation—such as driving the cytoplasmic-to-nuclear translocation of endocrine NRs—and remodels its associated co-regulatory complexes, thereby reprogramming transcription (4, 5). This core mechanism is finely tuned by post-translational modifications like phosphorylation and ubiquitination, which integrate signals from cell surface signalling pathways, allowing for intricate crosstalk (6). Thus, NRs act as integrative hubs, combining direct metabolic cues with broader cellular information to coordinate complex physiological responses.

In the realm of steroid receptors, a compelling brief report by Silva Chaves et al. addresses a critical intersection in physiology: the interplay between redox signalling and neuroendocrine homeostasis. The authors build upon their previous finding that antioxidant supplementation can paradoxically lead to hyperactivity of the hypothalamus-pituitary-adrenal (HPA) axis (7). Their new work, which probes the role of the Nrf2/haem oxygenase (HO)-1 pathway, represents a significant step in understanding this phenomenon. In a field where “antioxidants” are often simplistically equated with health, their data demonstrate that N-acetylcysteine (NAC) induces hypercortisolism by downregulating the glucocorticoid (GC) receptor (GR) in the pituitary and upregulating adrenal steroidogenic machinery (7).

The key novelty of this study (Silva Chaves et al.) lies in its mechanistic insight. The authors hypothesise that activating the Nrf2/HO-1 pathway with CoPPIX could counteract NAC-induced hypercortisolism. They demonstrate that CoPPIX not only reverses the hypercortisolism and adrenal enlargement but, more critically, restores GC negative feedback. They show that NAC disrupts the balance of GR isoforms in the pituitary by downregulating Nrf2, skewing expression towards the dominant-negative GRβ over the active GRα. CoPPIX rectifies this imbalance, thereby repairing the HPA axis’s ability to self-regulate. This work directly links redox signalling to endocrine feedback loops, suggesting that the Nrf2/HO-1 pathway is crucial for HPA axis homeostasis and that its suppression by certain antioxidants may underlie their adverse effects.

Shifting to pathology, a review by Hiltunen et al. re-examines the role of the GR in oncology. While initially recognised for its capacity to mitigate chemotherapy side effects (8), GR has emerged as a central and paradoxical player in prostate cancer, the classic androgen receptor (AR)-driven malignancy. As the review details, the crosstalk between GR and AR provides a key escape route for tumours evading AR-targeted therapies, with GR adeptly substituting for the inhibited AR to drive disease progression. Conversely, GR has also been shown to act as a tumour suppressor in certain contexts, which sustains the use of GCs as an adjuvant therapy. The authors emphasise the importance of understanding this duality of GR, considering cancer stage and specific cell types within the prostate tumour microenvironment. This review provides a framework for designing next-generation therapies that, strategically inhibit the detrimental actions of GR while preserving its beneficial effects.

The participation of NRs in regulating immune-endocrine responses is also critical in infectious diseases. Pérez et al. use Tuberculosis and Chagas Disease as key examples, highlighting how pathogens instigate a state of chronic inflammation and divert the host’s signalling pathways to their advantage. This dysregulation, mediated by ligand-dependent NRs, moves beyond a simple immune response to encompass broader metabolic and hormonal disorders. Unravelling the specific functions of NRs in these contexts is therefore paramount, offering a molecular lens through which to view pathogenesis and pioneer novel therapeutic strategies for these enduring global health challenges.

This concept is powerfully illustrated by new research into GR in Chronic Chagasic Cardiomyopathy (CCC). The study by González et al. reveals a clear dysregulation in GR signalling pathways. Their work shows that while the GR-α isoform is normally expressed in immune cells, CCC patients exhibit a significant imbalance in glucocorticoid signalling, marked by an increase in the enzyme 11β-HSD1 and an altered ratio between key inflammatory cytokines (IL-6, IFNγ) and the protective, glucocorticoid-regulated gene *TTP*. Furthermore, within the heart tissue itself, the presence of GR directly correlates with the severity of inflammation and cardiac hypertrophy. These findings demonstrate that the inflammatory damage in CCC is strongly linked to a failure in the body’s natural anti-inflammatory systems, directly implicating GR expression and function in the disease’s pathophysiology. This offers a compelling molecular mechanism for the observed chronic inflammation.

Together, the works presented in this Research Topic underscore that targeting specific NRs is not merely a theoretical pursuit but a promising therapeutic avenue. They offer a molecular key to mitigating the chronic inflammation that defines a range of global health challenges.
